# Supplementary material for: Contribution of own food production to household dietary diversity and nutrient intake adequacy among refugee households in Palorinya, northwestern Uganda
Source: PLoS One. 2026 Mar 26;21(3):e0345810. doi: 10.1371/journal.pone.0345810 (PMC13020840; doi:10.1371/journal.pone.0345810)
Supplement: Attachment — Submitted filename: Response to Reviewers.docx [file pone.0345810.s003.docx]

**Response to Reviewer Comments**

**Dear Editor-in-Chief,**

We thank you and the reviewers for the valuable comments which have helped to improve the quality of the manuscript. We have addressed all the reviewer comments.

**Response to Reviewer #1:**

Comment: You have presented an interesting study. However, your methodology is inconsistently described and not sufficiently referenced. You are reporting to have used the HDDS- based on which reference?

**Response: We are grateful for this comment. We have now clarified that the household dietary diversity score (HDDS) was assessed following guidelines from previous studies. These references have been explicitly cited in the “Variables and Measurements” section [Ref #: 37 & 38].**

Comment: It is also not clear why you aggregrated tht food groups to 9 food groups.Which ones were merged?

**Response: We thank you for this comment. We have explained in “Variables and Measurements ” section that although FAO recommends 12 food ,this study used nine food groups because sugar/sweets, fats and oils were not included as they contribute a low amount of nutrients. Moreover, there is no international consensus on which food groups to include in the calculation of HDDS. Supporting references [Ref #: 12 & 39] have also been included to justify the adaptation.**

Coment: Apart from this: you wrote you applied a 24h recall but then you used an FFQ?

**Response:** **We thank you again, however, we have clarified that the 24-hour dietary recall was used to assess HDDS, while annual food production and consumption data were used for nutrient intake estimation. This study did not use food frequency questionnaire (FFQ) which is based on 7 day recall which is used for calculation of food consumption score.**

Comment: In addition you are presenting the results as yearly intake based on one 24h recall ignoring any seasonal changes in availability, production and affordability.

**Response:We appreciate this comment. We recognize that using a single 24-hour recall for dietary diversity does not capture seasonal variation. To address this issue, we have now acknowledged it in the “Strength and Limitations” section. The text now reads that dietary intake and own food production are subject to seasonal fluctuations, therefore, a single 24-hour recall employed in this study only provides a snapshot of current dietary diversity and does not capture habitual or seasonal dietary patterns which could influence dietary diversity and nutrient adequacy outcomes.**

**Response to Reviewer #2:**

Comment: A 24 hour recall on diet diversity is insufficient to determine HDD.- Suggest indicate this strongly and maybe call it current diet diversity - and include this as one of the limitations. It might be also seasonal.

**Response: We thank you for this comment. We have revised the manuscript to refer to the outcome as “current household dietary diversity” and added a statement in the “strength and limitations” section explicitly acknowledging that a single 24-hour recall provides only a snapshot. The text now reads that dietary intake and own food production are subject to seasonal fluctuations, therefore, a single 24-hour recall employed in this study only provides a snapshot of current dietary diversity and does not capture habitual or seasonal dietary patterns which could influence dietary diversity and nutrient adequacy outcomes.**

Comment: There might also be a recall bias in terms of validity - discuss reliability, plausibility and limitations in this case.

**Response: Again, thank you for this comment. We have discussed recall bias as a potential limitation in the strength and limitation section of the manuscript. The text reads: “Limitations include the possibility of recall bias for self-reported responses during the survey without any means of validating the responses, thus influencing the validity of the findings.”**

Comment: The sample only includes one settlement area - and generalisation for the whole of Uganda is thus ungrounded.

**Response: We are grateful for this observation. This issue was as equally well acknowledged in the strength and limitation section of the manuscript that reads: “Our analysis did not use a nationally representative sample, so the findings are not likely generalizable to the entire country and other similar settings.”**

Comment: Is the statement of 0% of households relating to Calcium intake measured or assumed? Please elaborate and explain, clarify in paper.

**Response: We had already clarified this in the “Variables and measurements” section , and the text reads “To assess nutrient intake adequacy, data were collected on estimates of annual crop food production and consumption of the households using sacks, basins, tins, and all the quantities converted into standard unit of kilograms and grams”. In the “Discussion” section, we elaborated that the extreme inadequate intake of calcium by the households could be due to the fact that animal products were eliminated while capturing data of the previous own food produce. Moreover, animal products especially fish, milk and milk products represent one of the rich sources of calcium hence this finding may not represent a clear picture of calcium intake of the househiolds in this settlement.**

Comment: Clarify the contribution of own production as well as nutrient intake and the adequecy thereof - also clearly make a distinction between the two.

**Response: We appreciate this comment. However our study examined the contribution of own food production to household hold dietary diversity and nutrient intake adequacy among households in a refugee setting. The study revealed that while own food production contributed highly to vegetable consumption, it did not significantly support adequate intake of other essential food groups such as animal-sourced products, fruits, and dairy. Moreover, nutrient intake adequacy from own food production was alarmingly low across all essential micronutrients as stated in the “conclusion” section.**

Comment: Add VIF values - how was this calculated?

**Response: We have added that mean Variance Inflation Factor (VIF) values in Tables 5 & 6. These values were computed using Stata’s community contributed *collin* command before regression.**

Comment: PLease explain unemployed.

**Response:We have clarified in the “Variables and measurement” section that “unemployed” refers to household heads not engaged in any formal or informal income-generating activity at the time of the survey, including farming, trading, or casual labor.**

Comment: Correct the following - casual labor table say 41.8% and text 48.1%

**Response: We have corrected the inconsistency. The correct proportion is 41.8% as shown in Table 1, and the text has been revised accordingly**.

Comment: PLease clarify - 95% fell short -are 5% adequate? proteien RDA - since table shows 4.8% - clearly explain.

**Response: We grateful for this observation. We have corrected this issue in the abstract. Instead of 95%, it is 95.2% for zinc and protein. The text reads: “Own food production contributed minimally to nutrient intake adequacy, with most households falling short of annual recommended dietary allowance (RDA) for calcium (100%), iron (95%), zinc and protein (95.2%), and energy (99%).”**

Comment: There are inconsistencies between own production/self production and household food production

**Response: We have standardized terminology across the manuscript. The term “own food production” is now consistently used throughout to avoid confusion**.

Comment:Add missing values on tables

**Response: N/A. We analysed data on 316 subjects, no missing data values were reported.**
